# CBD: Coffee Beans Dataset

**DOI:** 10.1016/j.dib.2025.111434

**Published:** 2025-03-03

**Authors:** Bipin Nair B．J, Abrav Nanda K．M, Shalwin A．S, V. Raghavendra

**Affiliations:** Department of Computer Science, Amrita School of Computing, Amrita Vishwa Vidyapeetham, Mysuru, Karnataka, India

**Keywords:** Coffee bean, Brightness, Contrast, Grayscale

## Abstract

The development of advanced coffee bean classification techniques depends on the availability of high quality datasets. Coffee bean quality is influenced by various factors, including bean size, shape, colour, and defects such as fungal damage, full black, full sour, broken beans, and insect damage. Constructing an accurate and reliable ground truth dataset for coffee bean classification is a challenging and labour intensive process. To address this need, we introduce the Coffee Beans Dataset (CBD) which contains 450 high-resolution images sampled across 9 distinct coffee bean grades A, AA, AAA, AB, C, PB-I, PB-II, BITS and BULK with 50 images per class. These samples were sourced from Wayanad, Kerala, reflecting the region's diverse coffee bean quality .This dataset is specifically designed to support machine learning and deep learning models for coffee bean classification and grading. By providing a comprehensive and diverse dataset, we aim to address key challenges in coffee quality assessment and improvement in classification accuracy. When tested using the EfficientNet-B0 model, the model achieved a high accuracy of 100%, demonstrating its potential to enhance automated coffee bean grading systems. The CBD serves as a valuable resource for researchers and industry professionals, promot-ing innovation in coffee quality monitoring and classification algorithms.

Specifications Table

**Table:** 

Subject	*Computer Vision and Pattern Recognition*.
Specific subject area	*Coffee bean quality assessment, classification.*
Type of data	Images,figures
Data collection	*A team of four individuals have been assigned to collect the dataset. Coffee beans were placed in the light box and a controlled lighting setup using LED lights. A mobile camera Samsung S20 FE was mounted onto a tripod and positioned to capture the picture. Each picture contains 50 number of coffee beans in it. Pictures of each grade are split into different groups with appropriate labels.*
Data source location	*Kerala, Wayanad,Mananthavady.*
Data accessibility	Repository name: Mendeley Data Data identification number: 10.17632/52877z55vr.1 Direct URL to data: https://data.mendeley.com/datasets/52877z55vr/1
Related research article	*Jayakumari, Bipin Nair Balakrishnan, et al. "Coffee bean graded. based on deep net models." International Journal of Electrical & Computer Engineering (2088–8708) 14.3 (2024).*http://doi.org/10.11591/ijece.v14i3.pp3084–3093*.*

## 1 Value of the Data

A Technical contribution

• This information is useful for developing coffee bean grading and classification can be done through using deep and machine learning algorithms.
• The data are useful for extracting geometric features and image appearance features which is useful for classification and grading tasks
  B Knowledge transfer
• The dataset comprises nine different grades of Arabica coffee beans, each showing unique characteristics useful for classification and quality assessment.
• It contains meaningful insights into the minute details of coffee bean grading, covering aspects such as bean size, shape, color, and fault detection.
• The data serves as a bridge between traditional coffee grading methods and modern data-driven approaches

## 2 Objective

Coffee is one of the most popular beverage consumed globally, with its flavour profile being significantly influenced by the origin, variety, and processing techniques of the beans [1]. Despite the popularity of coffee effectively determining bean quality remains a challenge particularly for roasting and pricing purposes. Traditional methods rely heavily on human judgment and visual inspection which can be inconsistent and subjective [2].

**Fig. 1:**
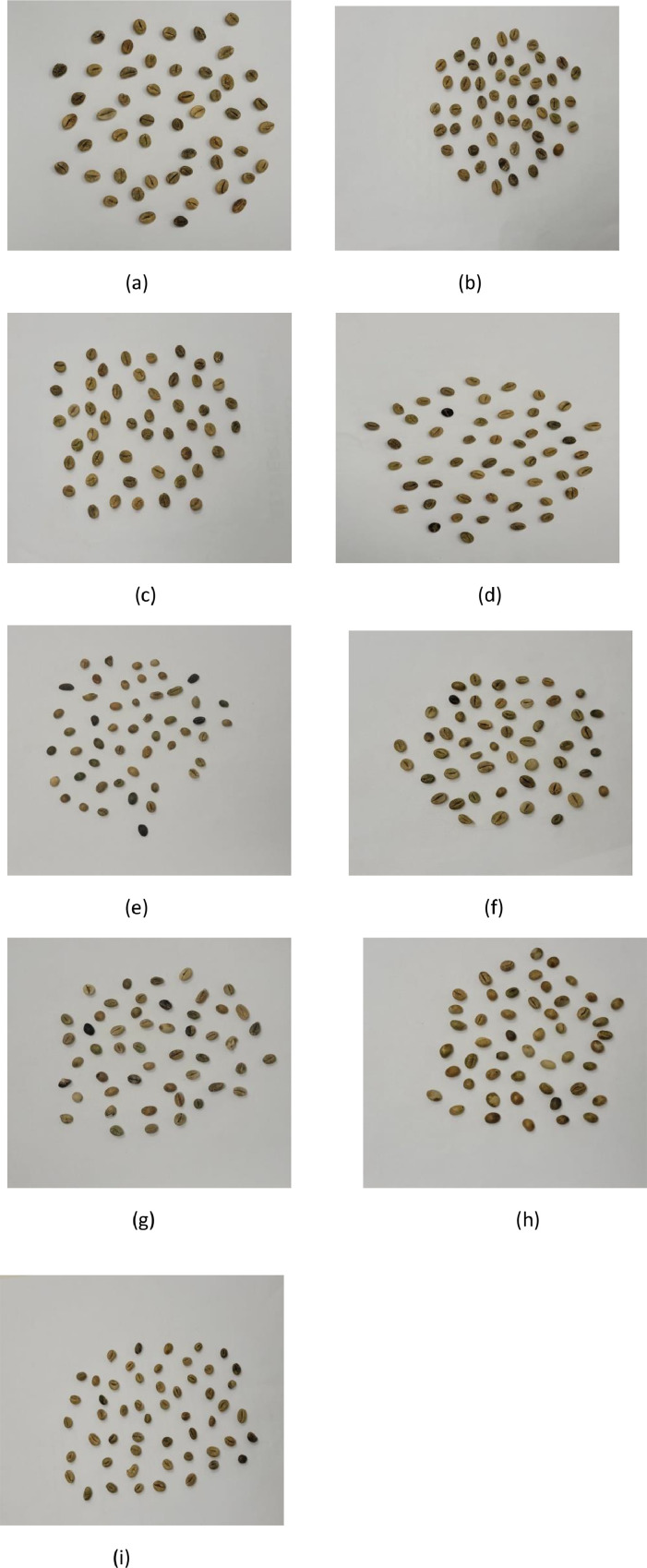
(a) Grade A (b) Grade AA .(c) Grade AAA (d) . Grade AB (e) Grade C. (f) Grade PB-I (g) Grade PB-II (h). Grade-BITS (i) Grade-BULK.

The primary objective of developing an image dataset of coffee beans is to enhance the accuracy and reliability of quality assessment methods. This dataset aims to address the complexities involved in coffee bean analysis by including images of beans with various imperfections such as cracks, breaks, moisture damage, discoloration, and deformities [3]. By creating a comprehensive dataset that captures these imperfections, the goal is to facilitate the development of advanced image analysis techniques including machine and deep learning models to improve the efficiency and objectivity of coffee bean quality assessment [4]. The dataset includes images representing nine distinct grades of Robusta coffee beans as illustrated in Fig. 1.

## 3 Data Description

This dataset is unique because there is no comprehensive and publicly available coffee beans image dataset specifically focused on the diverse coffee beans grades sourced from the Wayanad region of Kerala [5], The image acquisition process was designed to overcome common limitations typically encountered in standard imaging setups, such as varied lighting conditions and inconsistent backgrounds. Images were captured under controlled lighting using a lightbox to ensure uniform quality across all samples, making the dataset highly suitable for real time coffee beans grading and machine learning applications.

**Fig. 2:**
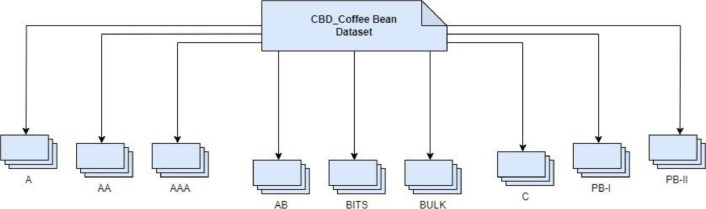
Folder structure of proposed dataset.

The coffee bean grades included in the dataset represent a wide range of quality levels, from high-grade to bulk, and were carefully handpicked with the assistance of local experts and coffee bean farmers from Kerala. A variety of coffee grades, each representing the distinct quality standards of the area can be observed in the dataset. Fig. 2. illustrates how the dataset is organized Additionally Table 1 offers detailed information on the coffee bean grades included in this dataset along with the corresponding image counts and quality categories.

**Table 1:** Dataset Collection: Category and source.

Sl.No	Category	No . of images	Source	Dataset repository
1	Grade-A	50	Kerala, Wayanad	doi:10.17632/52877z55vr.1
2	Grade-AA	61	Kerala, Wayanad	doi:10.17632/52877z55vr.1
3	Grade-AAA	50	Kerala, Wayanad	doi:10.17632/52877z55vr.1
4	Grade-AB	50	Kerala, Wayanad	doi:10.17632/52877z55vr.1
5	Grade-C	51	Kerala, Wayanad	doi:10.17632/52877z55vr.1
6	Grade-PB-I	51	Kerala, Wayanad	doi:10.17632/52877z55vr.1
7	Grade-PB-II	50	Kerala, Wayanad	doi:10.17632/52877z55vr.1
8	BITS	51	Kerala, Wayanad	doi:10.17632/52877z55vr.1
9	BULK	50	Kerala, Wayanad	doi:10.17632/52877z55vr.1

**Fig. 3:**
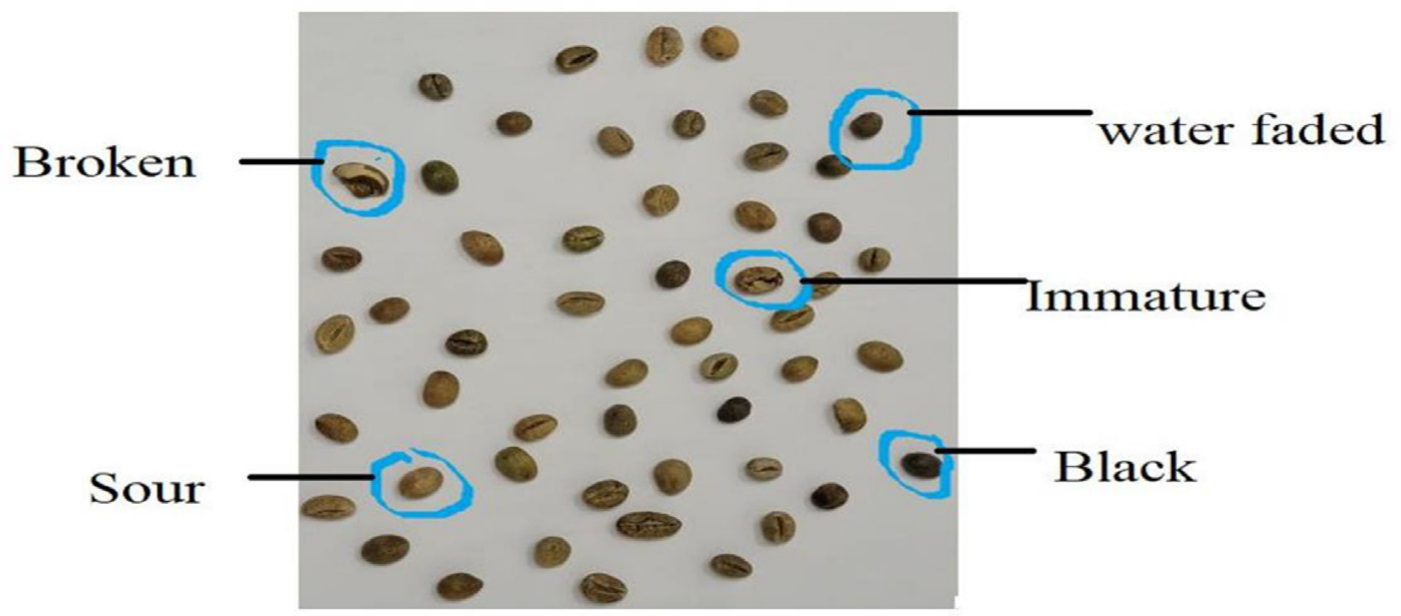
Various Defects in beans.

The Coffee Bean Classification Dataset (CBCD) represents a significant advancement in technology for automated coffee bean quality assessment. This labelled dataset addresses the limitations of previous datasets by incorporating real-world variations of coffee beans. The CBCD includes images of all nine quality grades, featuring examples of broken beans and wet beans Fig. 3.

## 4 Experimental Design, Materials and Methods

### 4.1 Data collection

The authors collected the coffee bean dataset directly from various sources within Wayanad, Kerala, India which makes the dataset unique and superior dataset. Working in collaboration with local coffee experts, farmers, and processors, the dataset was carefully assembled to capture the diversity and quality of coffee beans produced in the area [6].

### 4.2 Data acquisition

**Fig. 4:**
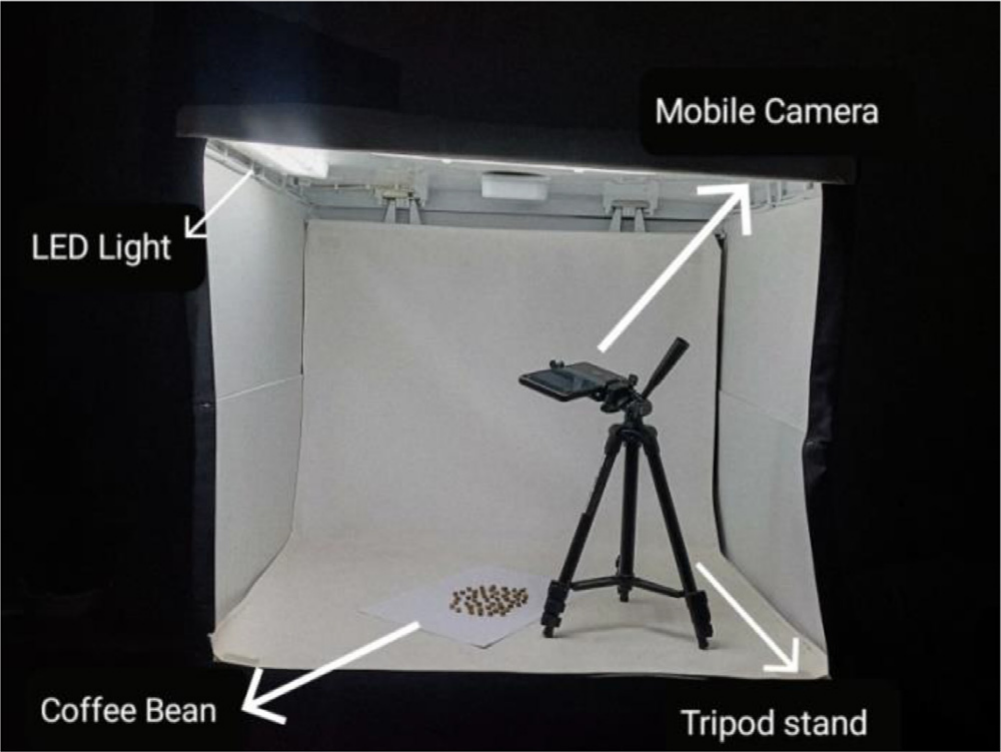
Dataset capturing setup.

All the images in the Coffee Beans Dataset were captured using a Samsung S20 FE smartphone, equipped with a 12 MP camera and mounted on a tripod (refer to Fig. 4). The coffee beans were placed inside a lightbox with controlled LED lighting to provide the necessary brightness while minimizing shadows. Each image consist 50 coffee beans arranged in a circular formation positioned upside down to ensure uniformity across all samples. This arrangement allowed for optimal visual clarity and consistent data capture. Utilizing this setup a total of 464 high-resolution images were collected encompassing nine distinct grades of Robusta coffee beans.

### 4.3 Data Pre-processing

The data pre-processing phase for the coffee dataset began by loading each image and converts it to grayscale to ensure uniform processing. The original images were then adjusted for brightness and contrast to enhance their visual characteristics. This adjustment was achieved by applying predetermined factors to modify the brightness and contrast levels. Following these adjustments, each image was resized to a standardized resolution of 256 × 256 pixels. In deep learning experiments all orientations were consider for calibration These pre-processing steps aimed to optimize the quality and consistency of the dataset for subsequent analyses. The final ground truth image was saved in either JPEG or PNG format. The present dataset is used in machine learning work got adequate accuracy same for deep leaning in an augmented form achieved good accuracy during the experiment we balanced the existing dataset.

**Fig. 5:**
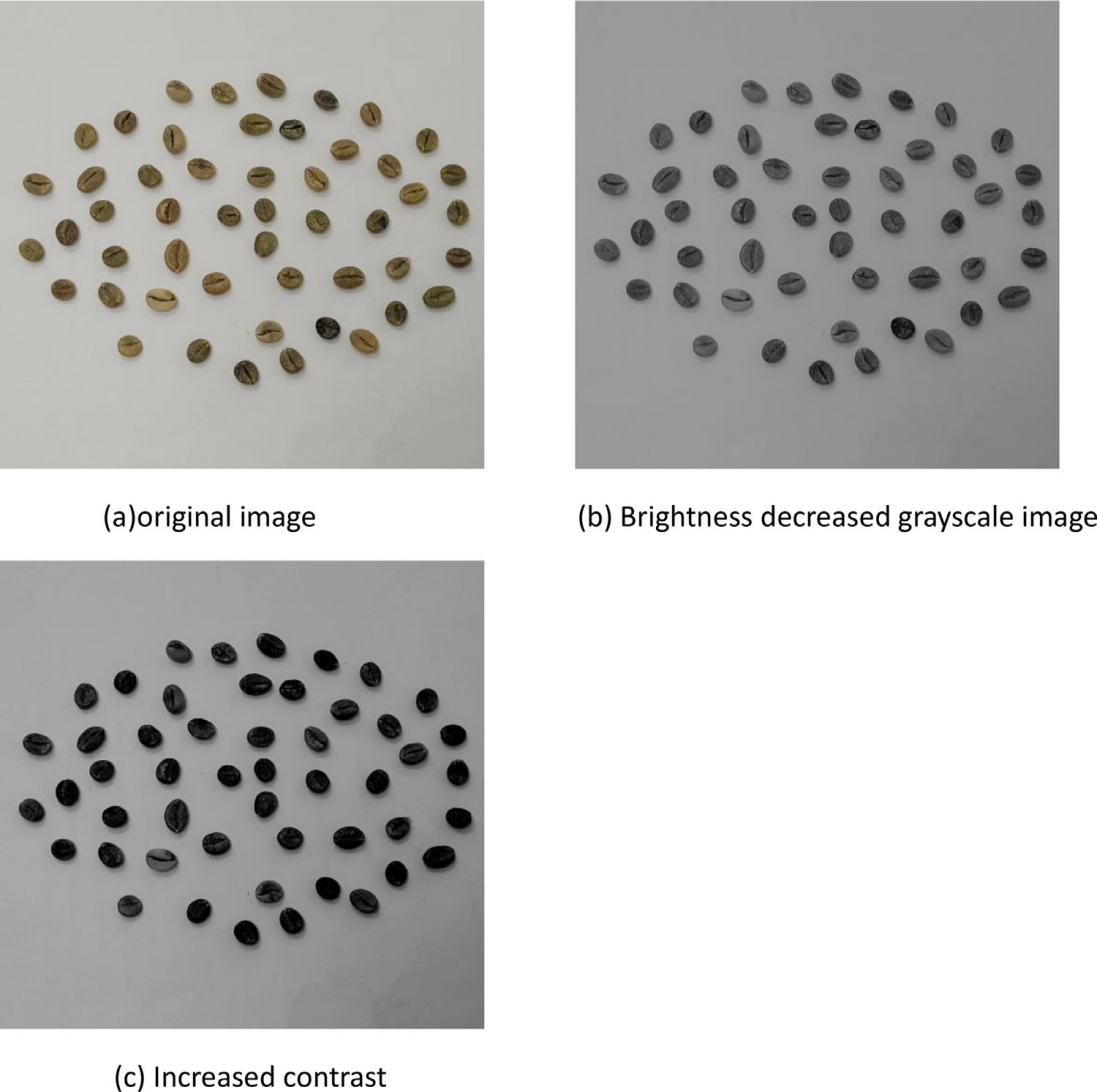
Displays ground truth images: (a) Original image (b) Brightness decreased gray scale image (c) Increased contrast image.

The experimental results shown in Fig. 5(a) to (c) illustrate the sequential stages of image processing applied to the coffee bean dataset. Initially, the original image is presented followed by the grayscale image with decreased brightness and increased contrast.

## Limitations

The coffee classification dataset, taken exclusively with a white background, may not accurately reflect real-world conditions with diverse backgrounds. The size of the dataset may be limited, limiting the diversity of samples and potentially reducing the representation of some coffee bean varieties. Inconsistent lighting can lead to contrast, and source biases may affect the dataset if the beans are not from diverse regions or if certain categories are underrepresented. In future quality grading can be increased by adding more classes as well as adulteration.

## Ethics Statement

This work does not include studies on animals, people or social media.

## CRediT authorship contribution statement

**Bipin Nair B．J:** Conceptualization, Supervision. **Abrav Nanda K．M:** Methodology, Formal analysis. **Shalwin A．S:** Writing – original draft, Validation. **V. Raghavendra:** Writing – review & editing, Writing – original draft.

## Data Availability

Mendeley DataCBD_Coffee Bean Dataset (Original data).
